# A comparison of McGrath MAC® and standard direct laryngoscopy in simulated immobilized cervical spine pediatric intubation: a manikin study

**DOI:** 10.1007/s00431-017-2909-9

**Published:** 2017-04-21

**Authors:** Marcin Madziala, Jacek Smereka, Marek Dabrowski, Steve Leung, Kurt Ruetzler, Lukasz Szarpak

**Affiliations:** 1 Department of Emergency Medicine, Medical University of Warsaw, Lindleya 4 Street, 02-005 Warsaw, Poland; 2 Department of Emergency Medical Service, Wroclaw Medical University, Wroclaw, Poland; 3 Department of Rescue and Disaster Medicine, Poznan University of Medical Sciences, Poznan, Poland; 4 Department of Outcomes Research, Cleveland Clinic, Cleveland, OH USA; 5 Department of General Anesthesiology, Cleveland Clinic, Cleveland, OH USA

**Keywords:** Airway management, Cervical immobilization, Videolaryngoscopy, Pediatric endotracheal intubation, Manikin study

## Abstract

Emergency airway management in children is generally considered to be challenging, and endotracheal intubation requires a high level of personal skills and experience. Immobilization of the cervical spine is indicated in all patients with the risk of any cervical spine injury but significantly aggravates endotracheal intubation. The best airway device in this setting has not been established yet, although the use of videolaryngoscopes is generally promising. Seventy-five moderately experienced paramedics of the Emergency Medical Service of Poland performed endotracheal intubations in a pediatric manikin in three airway scenarios: (A) normal airway, (B) manual in-line cervical immobilization, and (C) cervical immobilization using a Patriot cervical extrication collar and using two airway techniques: (1) McGrath videolaryngoscope and (2) Macintosh blade in a randomized sequence. First-attempt intubation success rate, time to intubation, glottis visualization, and subjective ease of intubation were investigated in this study. Intubation of difficult airways, including manual in-line and cervical collar immobilization, using the McGrath was significantly faster, with a higher first-attempt intubation success rate, better glottic visualization, and ease of intubation, compared to Macintosh-guided intubation. In the normal airway, both airway techniques performed equal.

***Conclusion:*** Our manikin study indicates that the McGrath may be a reasonable first intubation technique option for endotracheal intubation in difficult pediatric emergencies. Further clinical studies are therefore indicated.

**Table:** 

**What is known** **:** • *Airway management in pediatrics is challenging and requires a high level of skills and experience. Cervical immobilization is indicated in all patients with any risk of cervical spine injury, but it significantly aggravates endotracheal intubation in these patients. Videolaryngoscopes have been reported to ease intubation and provide better airway visualization in the regular clinical setting.*
**What is new:** • *The McGrath is an easy-to-use and clinically often used videolaryngoscope, but it has never been investigated in pediatrics with an immobilized cervical spine. In the normal airway, the McGrath provided better airway visualization compared to Macintosh laryngoscopy. However, better visualization did not lead to decreased time to intubation and a higher success rate of the first intubation attempt. In difficult airways, the McGrath provided better airway visualization and this led to faster intubation, a higher first-attempt intubation success rate, and better ease of intubation compared to Macintosh-guided intubation.*

## Introduction

As part of the “ABC” approach of advanced trauma life support, airway management is a crucial part of resuscitation of a critically ill child [5, 19]. Pediatric advanced life support guidelines focus on early oxygen administration and endotracheal intubation when “respiratory effort is inadequate, airway patency is compromised, or coma is present” [5]. Although widely discussed, endotracheal intubation is still considered the method of choice to secure the airway, enable oxygenation and ventilation, and protect from pulmonary aspiration of gastric content [11, 17]. Endotracheal intubation might be challenging, and the success rate mostly depends on personal skills and experience [7, 16, 28]. Children are generally considered more difficult to intubate and are at greater risk of failed intubation and complications [10]. The reported success rate of endotracheal intubation for children ranges between 33 and 95% and depends on the provider’s experience and the clinical setting [10, 21, 22]. Repeated intubation attempts delay oxygenation and increase the risk of adverse events [8, 11].

Almost 80% of pediatric spine injuries affect the cervical spine, and mortality of pediatric spine injuries is high [1]. Cervical spine immobilization is crucial in all patients with the potential risk of any cervical spine injury, as any movement of the injured cervical spine might cause secondary neurological complications. Airway management using direct laryngoscopy in patients with an immobilized cervical spine is challenging and sometimes even impossible [3]. Videolaryngoscopes have been introduced into clinical practice to increase visualization of the airway and ultimately ease endotracheal intubation [23]. However, the best device for managing the airway in pediatric patients with an immobilized cervical spine has not been determined yet.

The McGrath MAC (McGrath; Aircraft Medical Ltd, UK) is a videolaryngoscope that consists of a moderate-curved Macintosh videolaryngoscope blade and a camera connected to the handle. Important advantages of the McGrath are that providers are usually already familiar with the Macintosh blade and furthermore it allows the option of either direct or indirect view of the glottis with one line of sight [14]. Previously, the McGrath was reported to be faster and enable a higher first-attempt intubation success rate in children, even during ongoing chest compressions [26, 27]. However, whether the McGrath is effective in children with difficult airways, such as those with cervical immobilization, is unclear.

Therefore, this study aimed to compare the first-attempt intubation success rate of the McGrath and direct laryngoscopy for emergency intubation in a pediatric manikin model with an immobilized cervical spine.

## Materials and methods

This study was designed as a randomized, crossover manikin trial, was conducted between November and December 2016, and was approved by the Institutional Review Board of the Polish Society for Disaster Medicine (approval no.: 11/12/2016/IRB).

### Study participants

Seventy-five paramedics with <5 years of experience in out-of-hospital emergency medical services (EMS) participated in the study. The paramedics had not been trained on any videolaryngoscope before participating in this study. Furthermore, all paramedics had limited experience in pediatric “real-world” direct laryngoscopy-guided intubations (ranging between 3 and 10 intubations). All paramedics were verbally informed and gave their written consent to participate in this trial.

### Study protocol

**Fig. 1:**
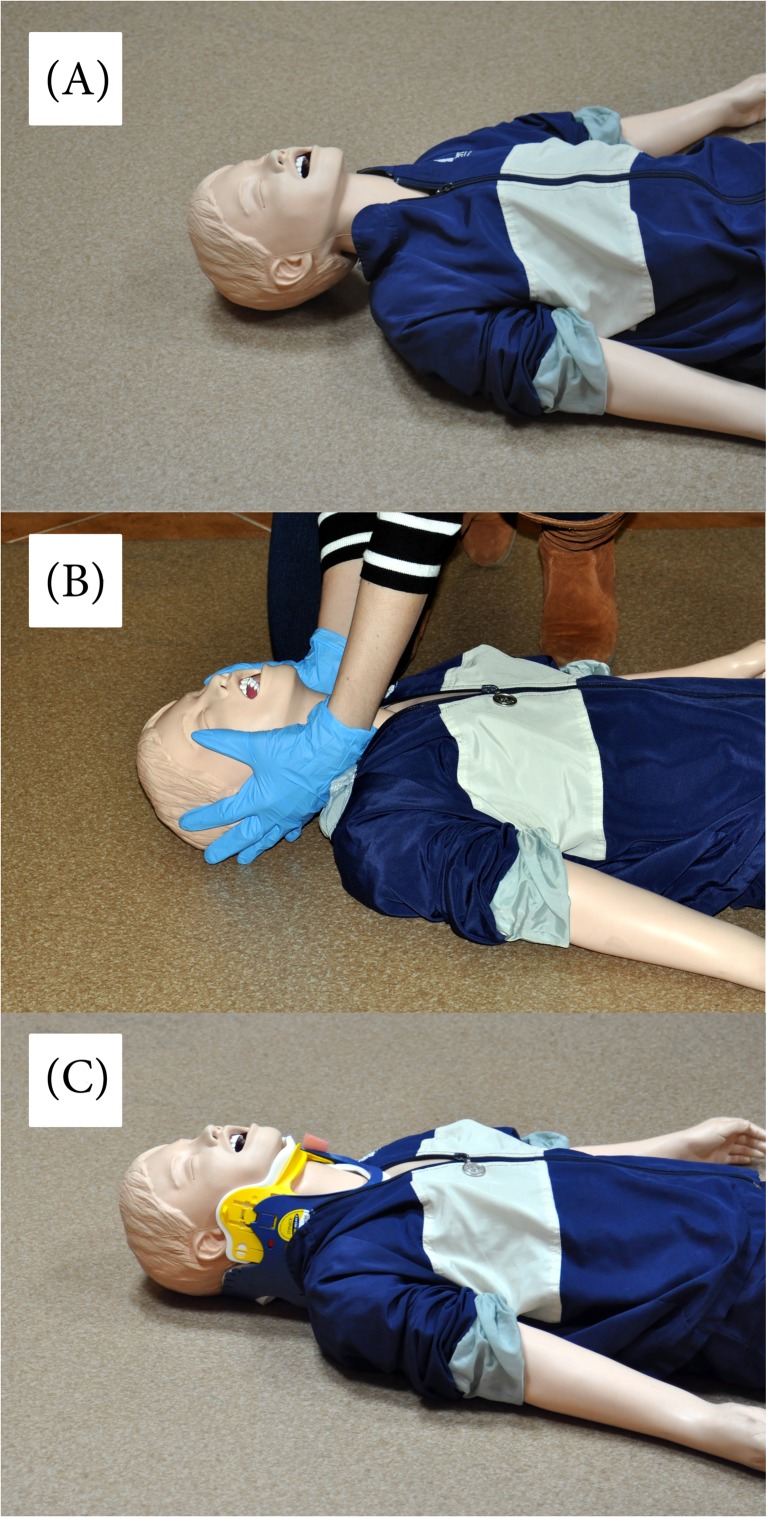
Intubation scenarios used in the study. **a** Scenario A—normal airway. **b** Scenario B—manual in-line cervical spine immobilization. **c** Scenario C—cervical immobilization using a standard Patriot cervical extrication collar

To simulate three different scenarios of endotracheal intubation, an airway manikin (MegaCode Kelly™; Laerdal, Stavanger, Norway, with a regular airway) was placed on the floor in a lighted room. All paramedics participated in three airway scenarios (Fig. 1):

1. Scenario A: normal airway without any cervical spine immobilization.
2. Scenario B: manual in-line cervical spine immobilization. Stabilization was performed by an independent instructor, not involved in airway management.
3. Scenario C: cervical immobilization using a standard Patriot cervical extrication collar (Össur Americas, Foothill Ranch, CA, USA), applied to the manikin’s neck by an independent instructor.

**Fig. 2:**
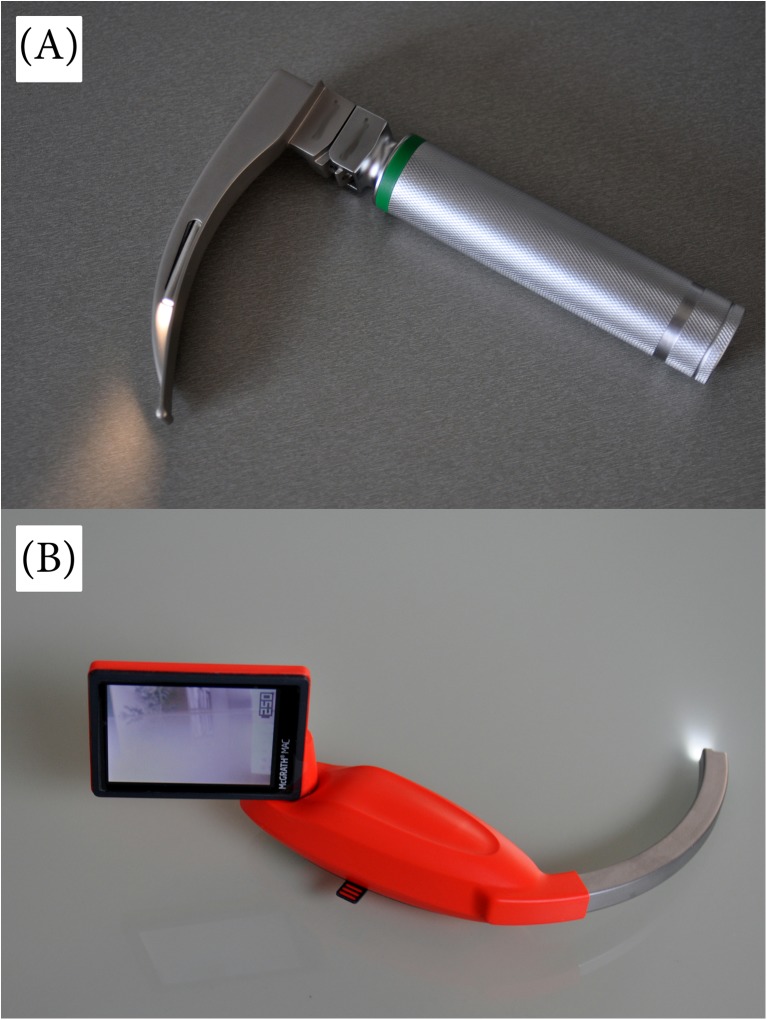
Laryngoscopes used for this study were **a** the standard Macintosh #2 laryngoscope and **b** the McGrath MAC laryngoscope

The following two airway techniques were used in this study: direct laryngoscopy using a Macintosh laryngoscope with blade size 2 (Mercury Medical, Clearwater, FL, USA) and the McGrath MAC with blade size 2 (McGrath; Aircraft Medical Ltd, UK) (Fig. 2).

All intubations were performed using a lubricated endotracheal (ETI) tube with a 5.0-mm internal diameter. The endotracheal tube was equipped with a hockey stick-shaped semirigid stylet for all intubations and the manikin, and the tube was wetted thoroughly with a lubricant.

After completing the first airway scenario, the paramedics had a 10-min break, before performing the second airway scenario. After finishing the second airway scenario, the paramedics again had a break lasting for 10 min. Afterwards, the paramedics performed the final airway scenario. The participants were not allowed to watch each other in order to avoid any teaching bias [29]. Each airway scenario was performed twice, once with direct laryngoscopy and once with the McGrath.

Before starting this trial, each paramedic participated in a 30-min audiovisual presentation covering all relevant aspects of human anatomy and basic principles of airway management with both devices used in this study. Afterwards, a researcher demonstrated both airway techniques, and the paramedics were allowed to perform endotracheal intubations in a 5-year pediatric airway trainer simulator (Gaumard® Scientific, Miami, FL, USA).

**Fig. 3:**
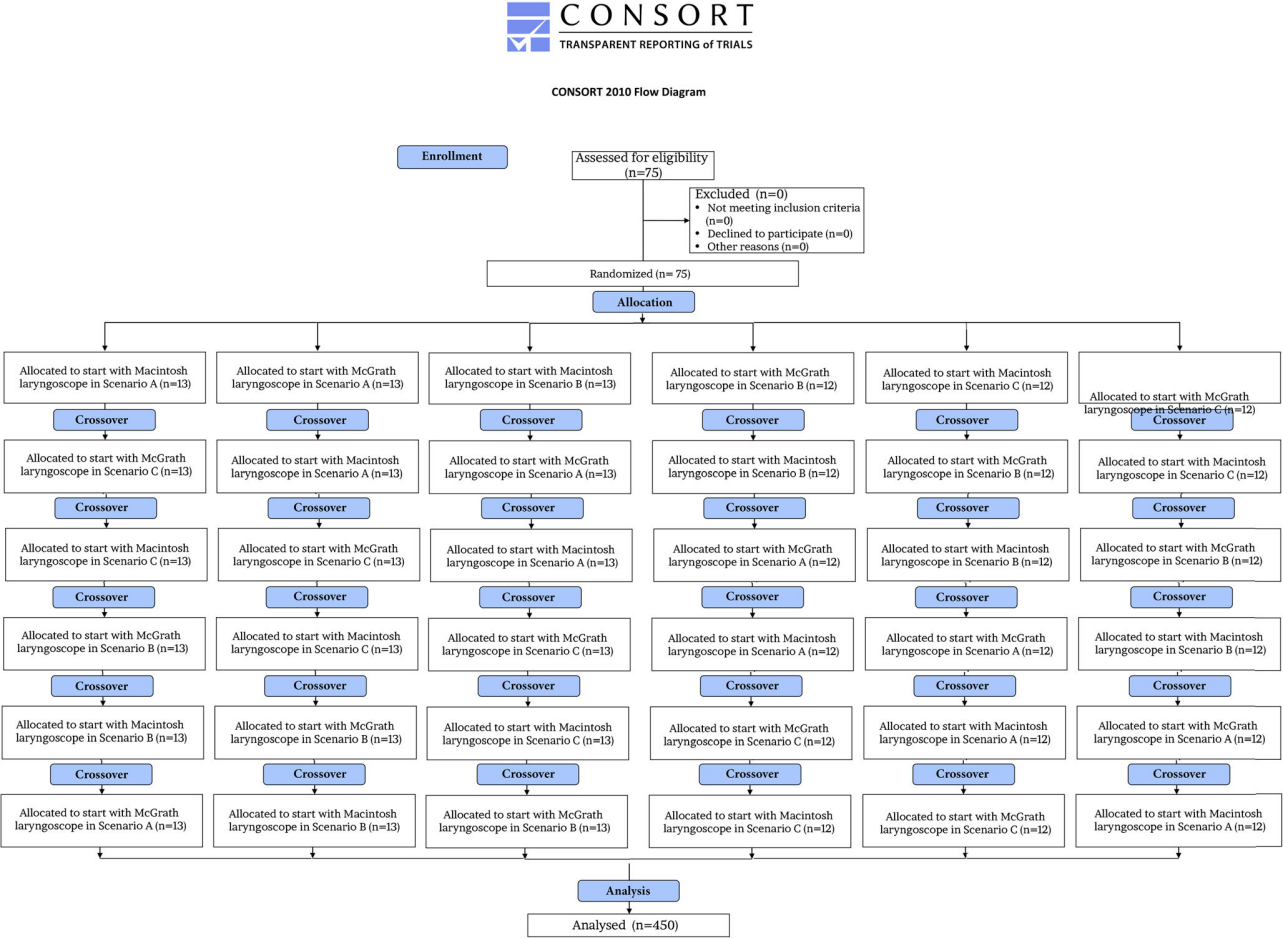
Flowchart of design and recruitment of participants according to the CONSORT statement

After the training session, all paramedics were randomly assigned to one out of six groups (two airway techniques and three airway scenarios) using the Research Randomizer software (www.randomizer.org) (Fig. 3). All intubations were performed in a MegaCode Kelly™ manikin. Intubation attempts were limited to a maximum of three attempts in each airway scenario, and each attempt was limited to a maximum of 60 s each. All paramedics were told that the patient is in critical circumstances and endotracheal intubation is indicated as fast as possible.

### Data collection

The primary outcome was the rate of successful placement of the endotracheal tube. The secondary outcomes were the time to intubation, quality of glottic view, and ease of intubation. Intubations were “successful” if the endotracheal tube was correctly placed within the manikin’s trachea, as confirmed by chest rise by a researcher. Intubations failed if the endotracheal tube was placed within the esophagus or intubation attempts lasted longer than 60 s. Time to intubation was defined as the time from insertion of the blade between the teeth until the first effective manual ventilation of the manikin’s lungs, as confirmed by one of the researchers. All intubation attempts were recorded with the sport camera HERO5 Black (GoPro GmbH, Munich, Germany), and time to intubation was analyzed afterwards.

Glottic view was evaluated by the paramedics by using the Cormack and Lehane classification system. At the end of each airway scenario, the paramedics were asked to rate ease of intubation using both airway techniques on a standard 10-point visual analog scale (VAS) ranging from 0 (very difficult) to 10 (very easy).

### Power calculation

Based on pilot data, the following assumptions were made to calculate the number of participants to be included: we proposed an alpha risk of 0.05 and a beta risk of 0.2. The success rate of the first intubation attempt in airway scenario C data amounted to be 60 versus 90% in direct laryngoscopy versus McGrath, respectively. Using a two-sided paired *t* test, we a priori estimated that at least 64 paramedics have to be enrolled. Based on an open call, we therefore included 75 paramedics in this study.

### Statistical analysis

The statistical software Statistica 13.1 (StatSoft, Tulsa, OK, USA) was used for statistical analysis. Percentages were used for qualitative variables and median with interquartile range (IQR) for quantitative variables. The occurrence of a normal distribution was confirmed by the Kolmogorov–Smirnov test. Nonparametric tests were used for the data that did not have a normal distribution. All statistical tests were two-sided. In order to compare the time needed to achieve endotracheal intubation, the Wilcoxon test for paired observations was used to determine the statistical difference for each group. The McNemar test was used to evaluate the differences in success of intubation. Glottic view grade and VAS score were all evaluated using the Stuart–Maxwell test. A *p* value <0.05 was considered significant.

## Results

A total of 75 paramedics (27 females; 36%) were enrolled in this study. All paramedics worked within an emergency medical service (EMS) in the out-of-hospital setting. The mean age was 27 [IQR, 24–30.5] years, and the mean work time experience was 2 [IQR, 0.5–4.5] years. The mean paramedic experience with direct laryngoscopy-guided pediatric intubation was 5 [IQR, 3–10] intubations.

### Time to intubation

**Fig. 4:**
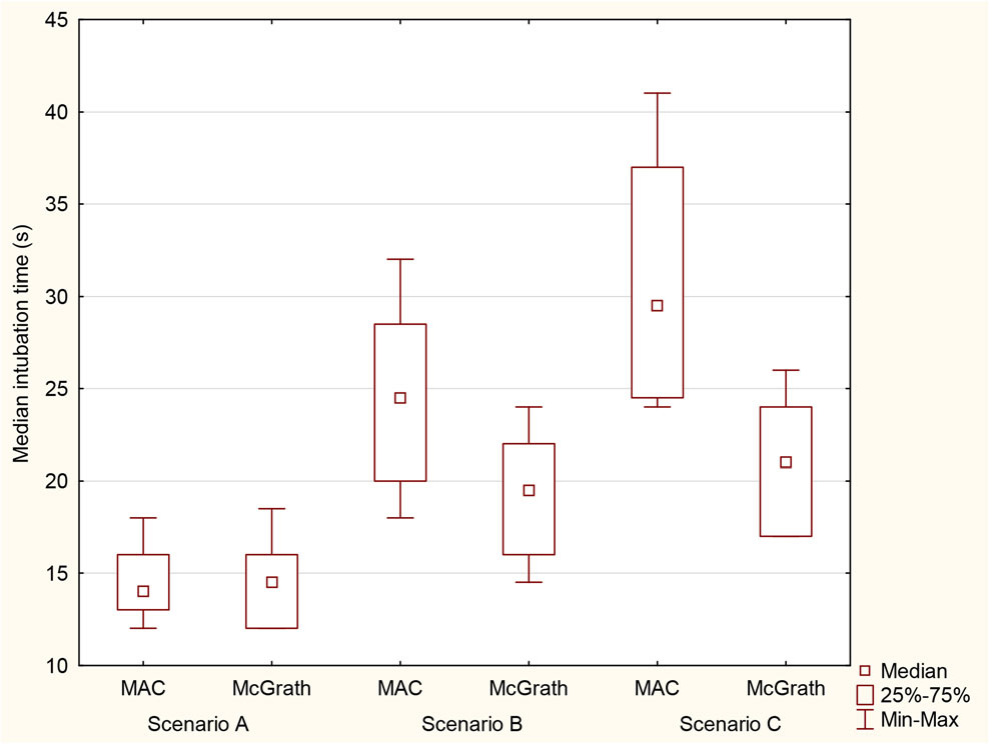
Median time (in seconds) required for endotracheal intubation with the two laryngoscopes in research scenarios

The median time to intubation using the Macintosh laryngoscope and McGrath during the examined emergency scenarios is shown in Fig. 4. In airway scenario A, there was no difference in the median time to intubation for either device (14 [IQR, 13–16] s for the Macintosh laryngoscope and 14.5 [IQR, 12–16] s for the McGrath). In airway scenario B, the median time to intubation was shorter in the McGrath (19.5 [IQR, 16–22] s), compared to direct laryngoscopy (24.5 [IQR, 20–28.5] s; *p* = 0.013). In airway scenario C, the median time to intubation using the McGrath was 21 [IQR, 17–24] s, compared to 29.5 [IQR, 24.5–37] s (*p* < 0.001) in the direct laryngoscopy group.

### Intubation success rate

In airway scenario A, all paramedics performed successful first-attempt intubation with both airway techniques. In airway scenario B, first-attempt intubation was successful in 72% in the direct laryngoscopy group, versus 99% in the McGrath group (*p* < 0.001; Table 1). The overall success rate in airway scenario B was 97% in direct laryngoscopy, versus 100% in the McGrath technique. In airway scenario C, the first-attempt intubation success rate was 93% in the McGrath, versus 45% in the direct laryngoscopy group (*p* < 0.001). The overall success rate was 100% in the McGrath group and 77% in the direct laryngoscopy group.

**Table 1:** Intubation success rate

Type of scenario	Parameter	Macintosh laryngoscope	McGrath laryngoscope	*p* value
Scenario A	First-attempt success rate	75 (100%)	75 (100%)	NS
	Overall success rate	75 (100%)	75 (100%)	NS
Scenario B	First-attempt success rate	54 (72%)	74 (99%)	<0.001
	Overall success rate	73 (97%)	75 (100%)	NS
Scenario C	First-attempt success rate	34 (45.3%)	70 (93%)	<0.001
	Overall success rate	58 (77.3%)	75 (100%)	<0.001

*NS* not statistically significant

### Glottic view grade

Glottis visualization is presented in Table 2.

**Table 2:** Glottic view grade

Type of scenario	Cormack and Lehane grade	Macintosh laryngoscope	McGrath laryngoscope	*p* value
Scenario A	I	65 (87%)	73 (97%)	NS
	II	10 (13%)	2 (3%)	
	III	–	–	
	IV	–	–	
Scenario B	I	14 (19%)	47 (63%)	<0.001
	II	36 (48%)	28 (37%)	
	III	25 (33%)	–	
	IV	–	–	
Scenario C	I	5 (7%)	32 (43%)	<0.001
	II	27 (36%)	43 (57%)	
	III	42 (56%)	–	
	IV	1 (1%)	–	

*NS* not statistically significant

### Ease of intubation

The visual analog scale score was significantly higher when the participants used the McGrath laryngoscope compared to when they used the Macintosh laryngoscope in all intubation scenarios: scenario A (*p* = 0.043), scenario B (*p* = 0.008), and scenario C (*p* < 0.001) (Table 3).

**Table 3:** Ease of intubation

Type of scenario	Macintosh laryngoscope	McGrath laryngoscope	*p* value
Scenario A	8.5 [IQR, 7–9]	9 [IQR, 8–9.5]	0.043
Scenario B	6 [IQR, 5–8]	8 [IQR, 7.5–9]	0.008
Scenario C	4 [IQR, 4–5.5]	7 [IQR, 6–7.5]	<0.001

*IQR* interquartile range

## Discussion

The main findings of this study are that there was no significant difference between the Macintosh and McGrath in terms of time to intubation, first-attempt intubation success rate, overall success rate, and glottic view in the paramedics’ hands during the normal airway scenario (scenario A). In contrast, significant differences were seen between these devices in the difficult airway scenarios, including manual in-line cervical immobilization and cervical collar immobilization. Our results are therefore consistent with previous studies, reporting videolaryngoscopy to be efficient in both pediatric and adult immobilized cervical spine settings [2, 4, 25].

In critically ill children, multiple intubation attempts substantially increase the risk of adverse events, including severe desaturation [15]. It was shown that pediatric intubation requiring two attempts has a threefold increased odds of desaturation below 80% compared to that requiring one attempt [12]. In our study, the McGrath outperforms the Macintosh by a large margin in both difficult airway groups in first-pass success rate. For the cervical collar scenario, the McGrath was significantly better than direct laryngoscopy for first-attempt success rate (93 vs. 45%). Failed first-attempt intubation also increases the risk of complications associated with repeated attempts [12].

The other important parameter we assessed is time to intubation. Several guidelines suggest that time to intubation should not exceed 20 s in newborns and 30 s in pediatrics [9]. As expected, the McGrath, due to its superior glottic view that is unimpeded by limited cervical motion and mouth opening, facilitated intubation in the manual in-line cervical immobilization group: decreased time to intubation by 20% and increased first-attempt success rate by 26%. An even more dramatic difference is seen in the cervical collar group: the McGrath decreased time to intubation by 28% and increased first-attempt success rate by 48%. Time to intubation is an important parameter in pediatric airway management. Time to intubation using direct laryngoscopy is notably exceeding 20 s in both difficult airway scenarios. In contrast, time to intubation using the McGrath was 19.5 and 21 s for in-line manual immobilization and cervical collar, respectively. This finding supports a previous finding, that time to intubation was about 20 s, even during ongoing chest compressions [27].

It was previously suggested that a difference of 5 s in time to intubation might be clinically significant [30]. The difference of median time to intubation in our difficult airway groups for the Macintosh and McGrath was 5 s for in-line immobilization and 8.5 s for the cervical collar, meaning the McGrath is likely to have positive clinically significant impact in real practice.

Our study contrasted with a recent meta-analysis by Sun et al. of 14 randomized trials comparing videolaryngoscopy and direct laryngoscopy in children, which showed that glottis visualization was improved with videolaryngoscopes but at the expense of increased time to intubation and failure rate [24]. A likely reason for the discrepancy is that, as noted by the authors, most randomized trials utilized experienced anesthetists as participants, which most likely biased their results. Additionally, experienced anesthetists were also more likely to be more experienced and more accustomed to direct laryngoscopy and may not yet mastered the eye–hand coordination required to manipulate the endotracheal tube through the vocal cords via guidance of the screen. Our study included relatively inexperienced paramedics. Although this might be a limitation of this study per se, this study reflects a real-world setting. Furthermore, all existing trials were conducted in children with normal airways; therefore, the advantages of videolaryngoscopes might be masked.

We also compared glottic visualization of these devices using the Cormack and Lehane classification system. We demonstrated that the McGrath resulted in significantly improved airway visualization in both difficult airway scenarios. For manual in-line immobilization, the majority (63%) of intubation attempts by the McGrath achieved a Cormack and Lehane grade of I, while a vast majority (81%) of Macintosh attempts achieved a grade of II–III. In the cervical collar group, similar improvements in glottic visualization were also seen with the McGrath. Our findings are reinforced by numerous previous studies, in both pediatrics and adults, that videolaryngoscopes improve glottic visualization [13, 20, 23, 24]. Very few studies, in contrary to our findings, reported that videolaryngoscopy degraded glottic visualization. For example, Riveros et al. suggested the GlideScope was associated with a poorer glottic view compared to direct laryngoscopy, but their results were confounded by limited blade size options for their pediatric patient [18]. Another study by Vlatten et al. reported the GlideScope was associated with a poorer view of the vocal cords compared to direct laryngoscopy in pediatric patients with in-line cervical immobilization [31]. The authors speculated that the GlideScope number 3 blade was too large, creating a picture too posterior to the glottic entrance. Another reason might be the inherent design advantages of the McGrath. The McGrath, with its more anterior position of the camera closer to the tip of the blade, provides a more anterior view of the larynx compared to the GlideScope, potentially aiming more directly at the glottic opening, especially in cases with severely limited cervical motion.

Importantly, all of our paramedics had no prior experience with any videolaryngoscopes and had minimal direct laryngoscopy-guided intubation experience. All paramedics were able to achieve improved views and quick intubation in pediatric difficult airways. We therefore conclude that videolaryngoscopy is easy to learn [6, 27]. Furthermore, the use of videolaryngoscopy is intuitive and also likely to ensure safe and effective intubations in any stressful environments such as in prehospital settings or in situations where pediatric airway experts are not available.

Our study has several strengths arising from its novelty and clinical relevance. To the best of our knowledge, our study is the first to compare direct laryngoscopy with the McGrath in a simulated pediatric airway with an immobilized cervical spine. It is impossible for us to predict whether the advantage of the McGrath will be translated into better clinical outcomes. However, we can speculate that decreased time to intubation will expedite oxygen delivery and an increased first-attempt success rate prevents airway complications associated with repeated laryngoscopy such as hypoxemia, aspiration, airway trauma, and bradycardia [15].

Our study has several limitations. Firstly, we utilized a pediatric manikin, which cannot simulate a real child and therefore may not reproduce precise intubation conditions of real patients. The use of manikins also allows us to achieve statistical power with a crossover design and reduce the inherent variability as with human subjects. In our present study, where cervical immobilization is simulated, it may not be ethical to purposefully induce cervical immobilization in noninjured children. Pediatric cervical injury is a relatively rare event, and thus, it would be challenging to conduct a true randomized controlled trial in the clinical setting. One further limitation is that we cannot entirely simulate difficult airway conditions, such as a bleeding airway and tongue edema and secretions, and airway structure movement with chest compressions and trauma directly due to the use of the devices cannot be evaluated. These points must be considered in adapting the McGrath in real pediatric patients.

## Conclusions

In conclusion, our manikin study demonstrated that the McGrath can assist paramedics to intubate in pediatric airway scenarios, including in-line immobilization and cervical collar, with significantly less time to intubation, higher first-attempt intubation success, better glottic visualization, and ease of intubation compared with the Macintosh. Based on our results, the McGrath may be a reasonable first emergency intubation technique option in pediatric patients with an expected difficult airway.

## Abbreviations

AHA: American Heart Association
CPR: Cardiopulmonary resuscitation
ERC: European Resuscitation Council
IRB: Institutional review board
IQR: Interquartile range
NS: Not statistically significant
VAS: Visual analog scale
